# Who got tested and who got sick? Sociodemographic inequalities in COVID-19 testing and hospitalization among 1.48 million individuals in Sweden

**DOI:** 10.1007/s10654-025-01321-x

**Published:** 2025-10-27

**Authors:** Olof M. Östergren, Emilie Counil, Arizo Karimi, Tove Fall, Jonas Björk, Karl Gauffin

**Affiliations:** 1 Department of Public Health Sciences, Stockholm University, SE 10693 Stockholm, Sweden; 2 Mortality, Health and Epidemiology, French Institute for Demographic Studies, Paris, France; 3 Department of Economics, Uppsala University, Uppsala, Sweden; 4 Molecular Epidemiology, Department of Medical Sciences, Uppsala University, Uppsala, Sweden; 5 Division of Occupational and Environmental Medicine, Lund University, Lund, Sweden; 6 Clinical Studies Sweden, Forum South, Skåne University Hospital, Lund, Sweden

**Keywords:** COVID-19, PCR-testing, Social inequalities, Register data

## Abstract

In the early stages of the COVID-19 pandemic, PCR testing served different purposes for individuals and for policy makers. Policy makers relied on testing for representative case numbers to track and mitigate the spread of the disease whereas individuals needed tests to protect themselves and others, or to travel or go work. Systematic differences in testing across population groups can bias case numbers, making it more difficult for policy makers to implement effective non-pharmaceutical interventions. We link records of 494 699 PCR-tests taken between 2020-07-01 and 2020-12-31 to individual records in several administrative registers for 1 480 126 working age individuals in the counties of Stockholm and Scania in Sweden. We estimate the likelihood of getting tested, test positivity rate and hospitalization risk by sex, household size, migration background, education, income and medical risk factors in the individual or in the household using regression models with age, occupation and neighbourhood as fixed effects. We find that testing behaviour vary independently by several demographic, socioeconomic and medical factors. Several groups that were at an elevated risk of being hospitalized for COVID-19, including men, individuals born outside Europe and those with low education, had low testing rates and high positivity rates. Numbers of confirmed SARS-CoV-2 infections reflect both infection rates and the testing behaviour of the population. To improve the utility of testing in future pandemics, policy makers may collect data on negative tests and dedicate part of the testing capacity for representative screening.

**Supplementary Information:**

The online version contains supplementary material available at 10.1007/s10654-025-01321-x.

## Introduction

Testing for infections is important for several reasons when societies and individuals attempt to manage pandemics [1, 2], especially in the early stages before vaccines are available. To limit the spread and consequences of infectious diseases and to understand transmission dynamics, it is important to identify who is infected. The effectiveness of non-pharmaceutical interventions such as lockdowns, mandatory face masks, contact tracing, and case isolation rely on accurate and up-to-date information on who is infected and who is not [1, 3, 4].

Policy makers are responsible for limiting transmission and mitigating negative consequences of the disease. This requires information on incidence and prevalence, but also information on vulnerable groups, that are either at a higher risk of being exposed or at a higher risk of developing severe illness if infected. In order to estimate the incidence and prevalence, as well as unbiased indicators of vulnerability, such as the case fatality rate, testing needs to be representative of the population [2].

However, individuals are unlikely to consider representativeness when deciding on whether or not to get tested. They may instead get tested to better protect their own health or that of their household members of or people in their care. [5] They may need a negative test to travel or to go to work [6]. In some groups, individuals are motivated to get tested because of social pressure, but in others, the stigma of being infected may instead deter from testing [5, 7]. Individuals can also avoid testing for fear of losing income if they test positive and cannot go to work, or if it is difficult or expensive to get tested [5, 8, 9].

Only a handful of studies have examined SARS-CoV-2 testing behaviour empirically. The results from these indicate that testing rates differed systematically in different population groups. Testing rates have been found to be lower among older persons [10–12], men [10, 11, 13], minority ethnic or racial groups [12, 14] and in areas with lower socioeconomic status (SES) in France [15], Sweden [16], Switzerland [17], the UK [12], and the US [18]. A Swedish study found that affluent groups were less likely to develop severe COVID-19 but more likely to test positive for SARS-CoV-2, likely because affluent groups got tested more frequently [19]. Similar patterns, with higher mortality but fewer detected cases in vulnerable areas, have also been found in the US [18] and Switzerland [17].

These studies suggest that testing behaviour differed between population groups and that groups that were at high risk of severe COVID-19 were less likely to get tested. However, since these studies either rely on area-level data [12, 15–18], are limited to one specific testing intervention [13], or focus primarily on other things than testing behaviour [18, 19], it is difficult to disentangle which factors are important and if they predict testing independently. The aim of this study is to simultaneously examine the role of individual-level demographic, socioeconomic and medical factors in the likelihood of getting tested for SARS-CoV-2, and the likelihood that the test is positive, using population-wide individual-level Swedish register data.

## Materials and methods

We link the records of positive and negative polymerase chain reaction (PCR) tests for the presence of SARS-CoV-2 to individual records in several administrative registers in Sweden. PCR testing was offered free of charge to the general public through the digital 1177 administrative system. We restrict the window of observation to the period from 2020-07-01 to 2020-12-31, when testing was widely available to the general public [20], while vaccines and over-the-counter antigen tests were broadly unavailable. Since specific testing policies varied between counties [20], we also restrict the geographic scope to two counties that used the 1177 system, Stockholm (2.39 million inhabitants) and Scania (1.38 million). Both counties comprise diverse populations living in both rural and urban areas and both offered the possibility to book an appointment at a dedicated testing site to get tested, either themselves or with assistance from a health professional. In addition, in Stockholm a home testing kit could be delivered and picked up by a courier. [20] We restrict the sample to working-age individuals since older persons had access to testing outside of the 1177 system, for example through nursing homes and at-home care services. We also exclude individuals who worked in health or residential care and were likely to have access to testing at work. We cross checked the positive tests in the 1177 system with SmiNet, a national database that contains all laboratory confirmed cases of SARS-CoV-2, and find that 78% of the cases recorded in SmiNet, had tested positive through the 1177 system in our analytical sample. The corresponding proportion was 51% among health care workers and 27% among persons older than 65 (Table S1, supplementary materials).

We obtain information on age, sex, household size, migration background, education, income and occupation at baseline. We also use information on health care use and prescription drugs from the last five years to identify medical risk factors in either the individual or the individual’s household members. We refer to the supplementary materials for a detailed description of the sample, registers and variable definitions. Our final sample comprise 1 480 126 individuals aged 30 to 64 who were alive and resident in Stockholm or Scania on 2020-07-01. During the follow-up, the individuals in the sample ordered a total of 494 699 tests, of which 65 471 were positive (Table 1).

**Table 1:** Descriptive statistics. Population numbers, number of tests and positive tests among persons aged 30–64, not employed in health or social care, resident in Stockholm and Scania, Sweden during 2020–07-01—2020–12-31

	Population		PCR tests		Positive tests	
	n	%	n	%	n	%
Sex						
Men	786 733	53.2	223 963	45.3	34 191	52.2
Women	693 393	46.8	270 736	54.7	31 280	47.8
Household size						
1	465 702	31.5	114 116	23.1	14 143	21.6
2	285 496	19.3	77 590	15.7	10 426	15.9
3	261 085	17.6	100 170	20.2	12 334	18.8
4 +	467 843	31.6	202 823	41.0	28 568	43.6
Migration background						
Native	852 469	57.6	325 448	65.8	38 833	59.3
Second generation	181 337	12.3	68 716	13.9	8802	13.4
Born in Europe	196 272	13.3	47 731	9.6	7365	11.2
Born outside Europe	250 048	16.9	52 804	10.7	10 471	16.0
Education						
Missing	39 634	2.7	4052	0.8	620	0.9
Compulsory	172 004	11.6	34 534	7.0	5904	9.0
Intermediate	550 529	37.2	167 130	33.8	25 007	38.2
Tertiary	717 959	48.5	288 983	58.4	33 940	51.8
Income quartile						
Low	388 025	26.2	77 466	15.7	10 702	16.3
Mid-Low	349 285	23.6	124 603	25.2	16 639	25.4
Mig-High	365 321	24.7	143 960	29.1	19 075	29.1
High	377 495	25.5	148 670	30.1	19 055	29.1
Medical risk factor (individual)						
No	1 014 013	68.5	340 362	68.8	46 125	70.5
Yes	466 113	31.5	154 337	31.2	19 346	29.5
Medical risk factor (household)						
No	1 078 094	72.8	345 853	69.9	44 715	68.3
Yes	402 032	27.2	148 846	30.1	20 756	31.7

Missing education is included as a covariate in the regression analyses but estimates for this group is not presented in the figures or tables

First, we use multivariate linear regression to estimate the mutually adjusted likelihood of taking at least one test during the period by sex, household size, migration background, education, income, individual medical risk factors, and medical risk factors in the household, with fixed effects for birth year and occupation (in 148 categories). While testing is likely to vary by occupation, it is not clear if differences by occupation are due to behaviour or because of occupational exposures or requirements from the employer. To account for unobserved neighbourhood characteristics including exposure to the virus and access to testing facilities, we also include small area fixed effects, defined as the demographic statistical area (DeSO) of residence (see supplementary materials for detailed information on DeSOs). We fit an additional model with the same right-hand specification estimating the test positivity, defined as the proportion of tests that were positive for each individual who took at least one test. These two measures provide complementary aspects of testing behaviour. For example, a high testing rate combined with a low positivity rate could indicate a low threshold for getting tested whereas a low testing rate combined with a high positivity rate could indicate a high threshold for getting tested.

Second, we examine if the groups that are more likely to test were also more likely to have a registered SARS-CoV-2 in SmiNet or to develop severe COVID-19. During the observation period, 80 339 individuals in the sample were registered in SmiNet and 2190 were hospitalized with COVID-19 as the main diagnosis. We fit four Poisson models that estimate relative risks of taking a test, testing positive, having a case registered in SmiNet and being hospitalized by sex, household size, migration background, education, income, and medical risk factors in the individual and in the household with fixed effects for age, occupation and DeSO. Relative risks facilitate the comparison of patterns in common (testing) and rare (hospitalization) events.

Finally, to get a sense of how much variation in testing behaviour can bias estimates of the true incidence of the disease at the local level, we compare the area-level correlation between the cumulative incidence of COVID-19 hospitalization, and testing propensity, test positivity, and laboratory confirmed cases of SARS-CoV-2 registered in SmiNet. Although it is likely that some groups seek care at later stages of the illness, the hospitalization rate likely vary less by individual behaviour than tests and cases.

## Results

**Fig. 1:**
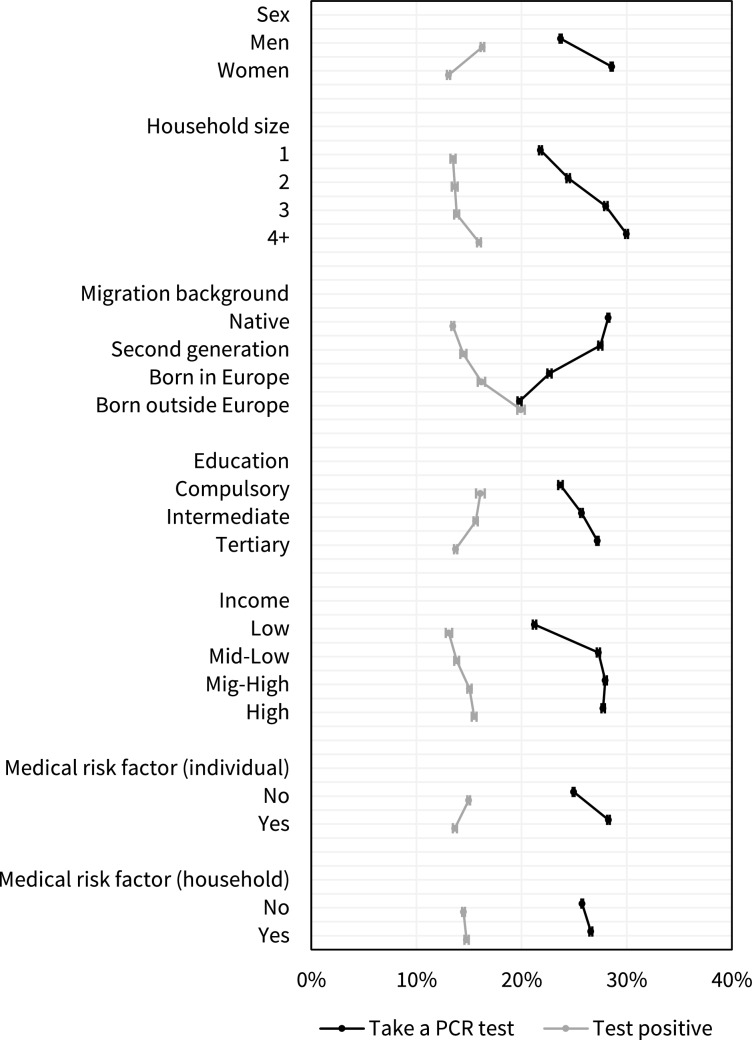
Predictive margins and 95% confidence intervals for taking a PCR-test and testing positive by demographic, socioeconomic and medical factors in Stockholm and Scania, Sweden, 2020-07-01—2020-12-31. The probabilities are estimated using mutually adjusted linear regression with fixed effects for DeSO, birth year and occupation in 148 categories. The probability of taking a test is estimated in the full analytical sample (n = 1 480 126) and the probability of testing positive is estimated in individuals in the sample that took at least one PCR test (n = 384 638). Predictive margins are estimated by fixing the variable of interest at a specific value for all individuals while keeping the other variables at their observed values, predicting the value of the dependent variable for all individuals and then averaging those predictions

The likelihood of getting tested and test positivity varied independently by all included factors (Fig. 1). In terms of sex, education, migration background and medical risk factors identified in the individual, the direction of association was inverted for testing propensity and positivity, meaning that the groups that ordered more tests were less likely to test positive. Men, individuals born outside Europe and those with low education or without a medical risk factor, were all comparatively less likely to order a PCR test, while at the same time having a higher relative risk of testing positive. For household size, income and medical risk factors identified in a household member, the associations were instead in the same direction, meaning that the groups that were more likely to get tested were also more likely to test positive.

Table 2 displays adjusted relative risks (RRs) for taking a test, having a case registered in SmiNet and being hospitalized with COVID-19, estimated in the full analytical sample, and the RRs for testing positive, estimated in the subsample of individuals who took at least one test. The patterns of hospitalization risk are overall similar to those observed for test positivity but are sometimes inconsistent with the patterns in having a case registered in SmiNet. There are no sex differences in detected cases in SmiNet and only modest gradients by migration background and education, where differences in hospitalization risk are prominent. Men, individuals in larger households, migrants and their descendants, individuals with low education and those with medical risk factors are all at an increased risk of hospitalization. Notably, individuals born outside of Europe were more than three times more likely to be hospitalized for COVID-19 compared to natives (RR: 3.25, 95% CI: 2.86, 3.69) and individuals with medical risk factors were more than four times more likely to be hospitalized compared to those without (RR: 4.19, 95% CI: 3.79, 4.63).

**Table 2:** Relative risks and 95% confidence intervals for taking a PCR test, testing positive, being registered with a case in SmiNet and being hospitalized because of Covid-19, Stockholm and Scania, Sweden, 2020-07-01—2020-12-31

	Take a PCR test	Test positive	SmiNet case	Hospitalization
Sex				
Men	1	1	1	1
Women	1.20 (1.19, 1.21)	0.80 (0.79, 0.81)	0.98 (0.97, 0.99)	0.53 (0.48, 0.58)
Household size				
1	1	1	1	1
2	1.16 (1.15, 1.17)	1.02 (0.99, 1.04)	1.11 (1.09, 1.14)	1.08 (0.95, 1.23)
3	1.30 (1.29, 1.31)	1.02 (0.99, 1.04)	1.27 (1.24, 1.30)	1.22 (1.06, 1.40)
4 +	1.37 (1.36, 1.38)	1.17 (1.14, 1.20)	1.54 (1.51, 1.58)	1.46 (1.29, 1.67)
Migration background				
Native	1	1	1	1
Second generation	0.98 (0.97, 0.99)	1.08 (1.05, 1.10)	1.07 (1.05, 1.09)	1.56 (1.35, 1.82)
Born in Europe	0.81 (0.80, 0.82)	1.20 (1.17, 1.23)	1.01 (0.99, 1.03)	2.00 (1.74, 2.29)
Born outside Europe	0.71 (0.70, 0.72)	1.47 (1.44, 1.51)	1.20 (1.18, 1.23)	3.25 (2.86, 3.69)
Education				
Compulsory	1	1	1	1
Intermediate	1.14 (1.12, 1.15)	0.98 (0.96, 1.01)	1.05 (1.02, 1.07)	0.88 (0.78, 0.98)
Tertiary	1.20 (1.18, 1.21)	0.86 (0.83, 0.88)	0.95 (0.93, 0.98)	0.73 (0.64, 0.84)
Income quartile				
Low	1	1	1	1
Mid-Low	1.35 (1.34, 1.36)	1.05 (1.02, 1.08)	1.37 (1.34, 1.40)	1.02 (0.90, 1.16)
Mid-High	1.39 (1.37, 1.40)	1.15 (1.12, 1.17)	1.52 (1.48, 1.55)	1.09 (0.94, 1.26)
High	1.38 (1.37, 1.40)	1.19 (1.16, 1.22)	1.56 (1.52, 1.61)	1.13 (0.96, 1.33)
Medical risk factor (individual)				
No	1	1	1	1
Yes	1.13 (1.12, 1.14)	0.91 (0.89, 0.92)	1.12 (1.11, 1.14)	4.19 (3.79, 4.63)
Medical risk factor (household)				
No	1	1	1	1
Yes	1.04 (1.03, 1.05)	1.02 (1.01, 1.04)	1.08 (1.07, 1.10)	1.17 (1.06, 1.29)

Relative risks are estimated using fixed-effects Poisson models. The models include all presented covariates as well as with fixed effects for age, DeSO and occupation. Relative risks for taking a test, being hospitalized with COVID-19 and having a case registered in SmiNet are estimated in the full analytical sample (n = 1 480 126) and the probability of testing positive is estimated in individuals in the sample that took at least one PCR test (n = 384 638)

**Fig. 2:**
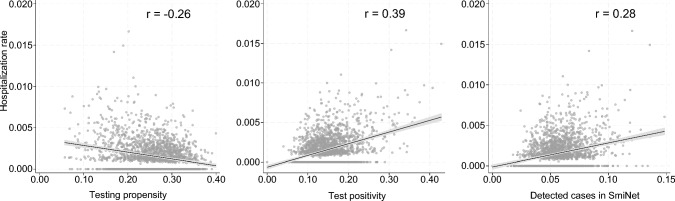
DeSO-level correlations between hospitalization rate and tests per capita ordered in 1177, test positivity rate and cases in SmiNet, Stockholm and Scania, Sweden, 2020-07-01—2020-12-31 (n = 2135). The estimates are based on the full population comprising 1.62 million individuals aged 30–64, 552 627 PCR tests ordered through 1177, 93 883 cases recorded in SmiNet, and 2468 hospitalizations for COVID-19

So far, the results have indicated that there are systematic differences in testing behaviour in the population. The pattern of testing behaviour, hospitalizations and cases recorded in SmiNet indicate that testing behaviour may have consequences for the ability to detect the true infection rate at the local level. Figure 2 shows that there is a negative correlation between neighbourhood-level testing propensity and hospital rates (r = − 0.26), but that the local positivity rate is positively correlated with the hospitalization rate (r = 0.39). There is also a positive, though substantially weaker, correlation between cases in SmiNet and hospitalization rate (r = 0.28).

The differences in testing behaviour presented in Fig. 1 and Table 2 are adjusted for DeSO, age and occupation as fixed effects. Using fixed effects allows us to adjust for detailed occupation and exact age without making assumptions about the functional forms of the associations but does not allow interpretation of the role of these factors. In the supplementary materials we present results from models where age (Fig S1), and occupation (Fig S2) are instead modelled as independent covariates. Younger persons ordered more tests and had lower test positivity rates (Fig S1). There are substantial differences in both testing propensity and positivity rate by the specific occupation but the testing propensity was overall higher and test positivity lower in occupations with higher skill levels while people in occupations with lower skill levels took fewer tests and had a higher test positivity rate (Fig S2). We also use an alternative approach where we model each medical risk factor separately (Fig S3). The increased testing propensity and lower test positivity among persons with risk factors can be attributed to a few conditions, in particular circulatory disease, respiratory disease and obesity, while several conditions, such as cancer, neuromuscular disorders and pregnancy, were associated with lower testing propensity despite being associated with an elevated risk of hospitalization. Differences in testing propensity were smaller when the condition was identified in a household member as opposed to the individual (Fig S3).

The infection rate as well as the strictness of non-pharmaceutical interventions increased in Sweden over the study period [21], which may have had different implications for the testing behaviour of different groups. We fit supplementary models where testing behaviour was allowed to vary by calendar month, finding that all groups had higher testing and positivity rates later in the study period (Fig S4). Still, differences in behaviour were similar throughout, though patterns were less pronounced due to the smaller number of tests taken in a single month, especially during the summer (Table S3).

We present the number of tests taken, positivity rates by number of taken tests as well as differences in getting tested by number of tests in Figure S5. Individuals taking multiple tests had lower positivity rates but repeated testing was uncommon; only around one percent of all individuals that got tested took more than three tests (n = 3888). The observed differences in testing behaviour tended to be more pronounced among individuals taking more than one test (Fig S5). These results indicate that repeated testing only made a modest contribution to the observed patterns. In addition, we fit a series of additional models where we estimate the probability of taking a test and test positivity in univariate models, in mutually adjusted models where we exclude DeSO fixed effects, and using separate indicators for each medical risk factor. Though the coefficient sizes varied somewhat, the patterns were strikingly similar across the different specifications (Table S4).

## Discussion

We find that testing behaviour depended on a range of factors. The marked difference in testing behaviour by sex, socioeconomic and migrantion status, after adjusting for occupation, age and likely exposure in the local area, suggest that testing is a social behaviour. Social norms encouraging the individual to get tested may be particularly strong in groups that are also economically and socially well equipped to adjust their lives to a positive test result. While the fear of serious illness may be equally strong across groups, fear of indirect consequences of a positive result, including the need to stay at home and loss of income, may deter some individuals from getting tested.

The decision of whether to get tested has consequences on disease transmission and severe morbidity in the population. In groups with lower testing propensity and higher positivity rates, there are likely more undetected cases which in turn can contribute to new infections and more severe cases. We find that men, individuals with low education and migrants were less likely to get tested and had higher positivity rates, indicating that the threshold for getting tested is higher in these groups. Previous studies have found that men [22, 23], individuals with lower socioeconomic status [19, 24, 25], and migrants [19, 26–28] have been especially affected by severe COVID-19, and we observe similar patterns. This indicates that the system for community testing in Sweden did not manage to reach vulnerable groups in the working-age population, and that this may have contributed to a higher burden COVID-19 in these groups through a higher rate of undetected cases.

Testing can be done for several reasons and serves several purposes, both for individuals and for policy makers. Our results indicate that the interests of the individual and policy makers may not always align. Taking a test improves the individual ability to protect themselves and others, but the high testing rates in population segments that have low risks of infection can lead to misrepresentative case numbers and make it more difficult to effectively mitigate the spread of the virus and a less accurate understanding of disease transmission and vulnerability [1, 29].

To improve the utility of testing in future pandemics, policy makers may, for example, dedicate part of the testing capacity to screen representative samples of the population. This would provide a less biased estimate of incidence and prevalence as well as a more appropriate population base to calculate vulnerability and case fatality. We furthermore find that the local test positivity rate has a stronger correlation with the incidence of severe COVID-19 cases than laboratory confirmed cases (Fig. 2). This finding indicate that policy makers can improve accuracy in monitoring and mitigation efforts if they were to collect data on both negative and positive tests, ideally along with other relevant data such as the reason for testing, response times and the method of testing. This would improve the ability of both individuals and health systems to monitor and limit the spread of disease in future pandemics.

This is the first study to use population-wide individual-level data to assess differences in COVID-19 testing behaviour by several types of potential predictors of testing. In doing so, we are able to bridge the literature on testing intentions and the limited literature on testing behaviour. Still, the study has several limitations. We are restricted to the administrative register data that are available to us. Some factors which we are unable to observe are likely to be important, such as trust and health seeking-behaviour [5], access to testing sites [8], experienced symptoms [14], and psychological characteristics [11]. Several of these are likely to correlate with some of the included factors, making it difficult to make precise interpretations of the processes that generate the observed patterns.

We are also limited to one system for testing. While 78% of all laboratory confirmed cases in our analytical sample were detected through the 1177 system (Table S1), we do not have information on the tests that were used to detect the remaining 22% of cases. These were detected outside of the system, for example through routine tests at hospital admissions or health care visits, tests taken for the purpose of travel certificates or outside of the county of residence. To the extent that some groups were systematically more likely to get tested through other systems, we may either over- or underestimate the observed differences in testing.

In conclusion, we find systematic differences in COVID-19 testing propensity in the population by demographic-, socioeconomic- and health status during early phases of the pandemic in two Swedish counties. Groups that are at high risk of developing severe COVID-19 are often the least likely to get tested, which can contribute to further disease transmission in these groups and exacerbate social inequalities in COVID-19. Numbers of confirmed infections reflect both infections but also systematic differences in testing behaviour in the population.

## Supplementary Information

Below is the link to the electronic supplementary material.

Supplementary file1 (PDF 340 KB)

## Data Availability

Access to register data for the purposes of this study was made possible through participation in the SWECOV project. The data comprises register data collected from several Swedish administrative registers. We are unable to publicly share the data on both ethical and legal grounds. To replicate or expand on this work, researchers may obtain an ethical permit and the same data material by contacting the specified register holders.
