# Supplementary material for: Who got tested and who got sick? Sociodemographic inequalities in COVID-19 testing and hospitalization among 1.48 million individuals in Sweden
Source: Eur J Epidemiol. 2025 Oct 27;40(12):1431–9. doi: 10.1007/s10654-025-01321-x (PMC12756271; doi:10.1007/s10654-025-01321-x)
Supplement: Supplementary file 1 — Supplementary file1 (PDF 340 KB) [file 10654_2025_1321_MOESM1_ESM.pdf]

Who got tested and who got sick?

Sociodemographic inequalities in COVID-19 testing and hospitalization among 1.48 million individuals in Sweden

SUPPLEMENTARY MATERIALS

Table S1. The proportion of all confirmed cases of SARS-CoV-2 registered in SmiNet that had an infection detected through 1177 system and through other testing channels in Stockholm and Scania, Sweden, 2020-07-01—2020-12-31.

	Analytical sample		Healthcare workers		Age 65+	
Detection system	n	%	n	%	n	%
1177	62 899	78.3	6839	50.5	3822	27.2
Other	17 440	21.7	6708	49.5	10 252	72.8

Note: During the pandemic, a laboratory that detected the presence of SARS-CoV-2 was required by law to report this to the Public Health Agency of Sweden that recorded them in the SmiNet database. The SmiNet database does not contain information on the date the test was taken and through which system. A detected infection will only register as a new case if the person had not tested positive in the preceding six months.

Registers and variable definitions

Where applicable, the Swedish names for registers, classification standards and agencies are provided in parenthesis.

Study sample

The population comprise all individuals alive and resident in Stockholm or Scania on 2020-07-01, identified through the Total Population Register (*Registret över totalbefolkningen, RTB*), Statistics Sweden (*SCB*). The sample is restricted to ages 30-64. In these ages, individuals are likely to have established independent households and rely on income from economic activity on the labour market. At older ages, individuals are more likely to get tested through other means, for example through at-home care or assisted living facilities. Individuals that worked in health care or residential services were likely to be tested at work and were excluded from the study. These were identified by being employed at workplaces classified as either Q86 (Human health activities) or Q87 (Residential care activities) according to the Swedish Standard Industrial Classification (*Standard för svensk näringsgrensindelning, SNI*) in the 2019 occupational register (*Yrkesregistret*), Statistics Sweden (*SCB*). We were not able to assess mid-year internal migration to account for changes in the county of residence during the follow-up, though these events are likely few given the short follow-up period. The final sample comprised n=1 480 126 individuals. Test positivity rate was estimated in a sub-sample defined as individuals in the full sample that took at least one PCR-test using the 1177-system during the observation period (n=384 638).

Dependent variables

PCR tests were defined as having a record of ordering at least one PCR test during the period 2020-07-01 to 2020-12-31 using the 1177 system. This register was obtained from Stockholm and Scania county councils via Inera, the company that operates the system. Inera is a private company owned by the association of local and regional authorities (*Sveriges Kommuner och Regioner, SKR*). Test positivity was defined as the proportion of the taken tests that were positive.

Hospitalization was defined as having at least one episode of inpatient care where Covid-19 was the main diagnosis, identified with codes U07.1 (COVID-19, virus identified) or U07.2 (COVID-19, virus not identified) according to the 10th revision of the International Classification of Diseases (ICD-10). Hospitalizations were observed using the National Patient Register (*Patientregistret*), the National Board of Health and Welfare (*Socialstyrelsen*). This definition excludes individuals that were hospitalized for other conditions than Covid-19, even if they were infected at the time of hospitalization. It also excludes individuals who were infected at the hospital.

Independent variables

Demographic factors were obtained from the total population register (*Registret över totalbefolkningen, RTB*), Statistics Sweden (*SCB*). These include, sex, age, household size and migration status. Age was modelled as a fixed effect, defined as the exact year of birth. In supplementary analyses, age was modeded as a set of dummy variables indicating exact age in years in 2020. Household size was defined as the number of individuals of any age residing in the

household. Migration status was categorized into *native* (born in Sweden with two Swedish-born parents), *second generation* (born in Sweden with at least one foreign-born parent), *born in Europe* and *born outside Europe*.

Information on education, occupation and income was obtained from the Longitudinal integrated database for health insurance and labour market studies, LISA (*Longitudinell integrationsdatabas för sjukförsäkrings- och arbetsmarknadsstudier, LISA*), which comprise of data collected from several registers including the educational register (*Utbildningsregistret*) and the tax-and income register (*Inkomst och Taxeringsregistret*), and is maintained by Statistics Sweden (*SCB*). Education in 2019 was categorized into *missing* (this category includes individuals that can be linked to the educational register, but the level of education is unknown), compulsory (SUN-2000 (*Svensk utbildningsnomenklatur 2000*) level 00—31, equivalent to ISCED-2011 (International Standard Classification of Education 2011) level 0—2), intermediate (SUN-2000 level 32—33, ISCED-2011 level 3), and tertiary (SUN-2000 level 41—64, ISCED-2011 level 4—6). Income was defined as average disposable household income in the period 2015—2019, divided into quartiles within each one-year birth cohort. Occupation was defined as the occupation held by the individual in 2019. It was modelled using 148 categories defined by the three first digits of the 2012 Swedish Standard Classification of Occupations (*Standard för svensk yrkesklassificering, SSYK*), or ten categories based on the first digit of the SSYK standard, which indicate the skill level of the occupation.

Medical risk factors were identified using the National Patient Register (*Patientregistret*) for both hospital care and specialized open care and the National Prescribed Drug Register (*Läkemedelsregistret*), both kept by the National Board of Health and Welfare (*Socialstyrelsen*). Throughout the pandemic, Swedish authorities identified pre-existing conditions that may lead to a higher risk of infection of SARS-CoV-2 or higher risk of severe COVID-19 if infected. The definition was constructed based several lists of conditions used by Swedish authorities at different stages of the pandemic. The list therefore includes conditions that may not necessarily be associated with an increased risk of infection or increased risk of severe consequences of an infection, but instead is an inclusive list of conditions that may have led individuals to believe that they were at an increased risk. The conditions were identified through diagnosis and treatment during episodes of inpatient care or visits to specialized outpatient care during 2015-2019, or during 2020, indicated by ICD-10 diagnosis or certain care measures defined by the KVÅ standard (*Klassifikation av Vårdåtgärder*) codes and/or purchases of prescribed medications in 2019 or 2020, indicated by the ATC (Anatomical Therapeutic Chemical) code. A person was classified as having a medical risk factor if any of the observed conditions were indicated in their medical history. A person was classified as having a household member with a medical risk factor if either of the included conditions were observed for any household member, regardless if that person was in the study sample. The included diagnoses, care measures and drugs are presented in Table S2.

We link the data to the geography database (*Geografidatabasen*), Statistics Sweden (*SCB*) to obtain the DeSO of residence. Sweden is divided into 5984 demographic statistical areas, DeSOs (*Demografiska statistikområden*), 2135 which are located in Stockholm or Scania. Each comprise between 700 and 2700 inhabitants and is defined by natural borders such as rivers, train tracks, forests and bigger roads. They also follow borders of municipalities and counties, which are the units at which social care and health care are organized within, respectively.

Table S2. Definitions of medical risk factors.

Condition	Criteria
Cancer	**ICD-10:** C*, Z85
	**KVÅ:** DT107, DT108, DT112, DT116, DT135
Diabetes	**ICD-10:** E10–E14
	**ATC:** A10
Dementia	**ICD-10:** F00–F02, F03.9, F10.7A, G30
	**ATC:** N06D
Psychological disorders	**ICD-10:** F20, F25, F30, F31
	**ATC:** N05AN
Alcohol abuse	**ICD-10:** F10, E24.4, G31.2, G62.1, G72.1, I42.6, K29.2, K70, K85.2, K86.0, Q86.0, Z71.4, Z72.1
	**ATC:** N07BB
Drug abuse	**ICD-10:** F11–F19
	**ATC:** N07BC
Diseases of the circulatory system	**ICD-10:** I10.9, I11-I13, I15, I20-I25, I48, I50, I60, I61, I62, I63, I64, I69, I70
	**ATC:** B01AC24, C01DA, C02 (excluding C02AC02), C03, C07, C08CA, C09, N02BA
Chronic respiratory disease	**ICD-10:** J40–J47, J60–J67, J68.4, J69, J70.1, J70.3, J84, J96.1, J96.8, J96.9, E84
	**ATC:** R03AK, R03AL, R03BA, R03AC12, R03AC13, R03AC18, R03AC19, R03CC12, R03BB04, R03BB05, R03BB06, R03BB07
Chronic liver disease	**ICD-10:** K70–K71, K72.1, K72.9, K73–K77
Kidney disease	**ICD-10:** I12, I13, N00–N08, N11, N14, N18–N19, Z99.2, E10.2, E11.2
Neuromuscular disorders	**ICD-10:** G10–G14, G20–G26, G30–G32, G35–G37, G70–G73, G80–G83
Obesity	**ICD-10:** E66
	**ATC:** A08
Rheumatoid arthritis	**ICD-10:** M05, M06, M08
Pregnancy (2020 only)	**ICD-10:** O*, Z33.9
Immunocompromised	**ICD-10:** D80–D89
Adrenal insufficiency	**ICD-10:** E27.1, E27.4
Organ transplants	**ICD-10:** Z94

Figure S1. Predictive margins and 95% confidence interval for taking a PCR-test and testing positive by age, Stockholm and Scania, Sweden, 2020-07-01—2020-12-31. Linear regression adjusted for sex, household size, migration background, education, income, medical risk factors, medical risk factors in the household and fixed effects for DeSO and occupation in 148 categories.

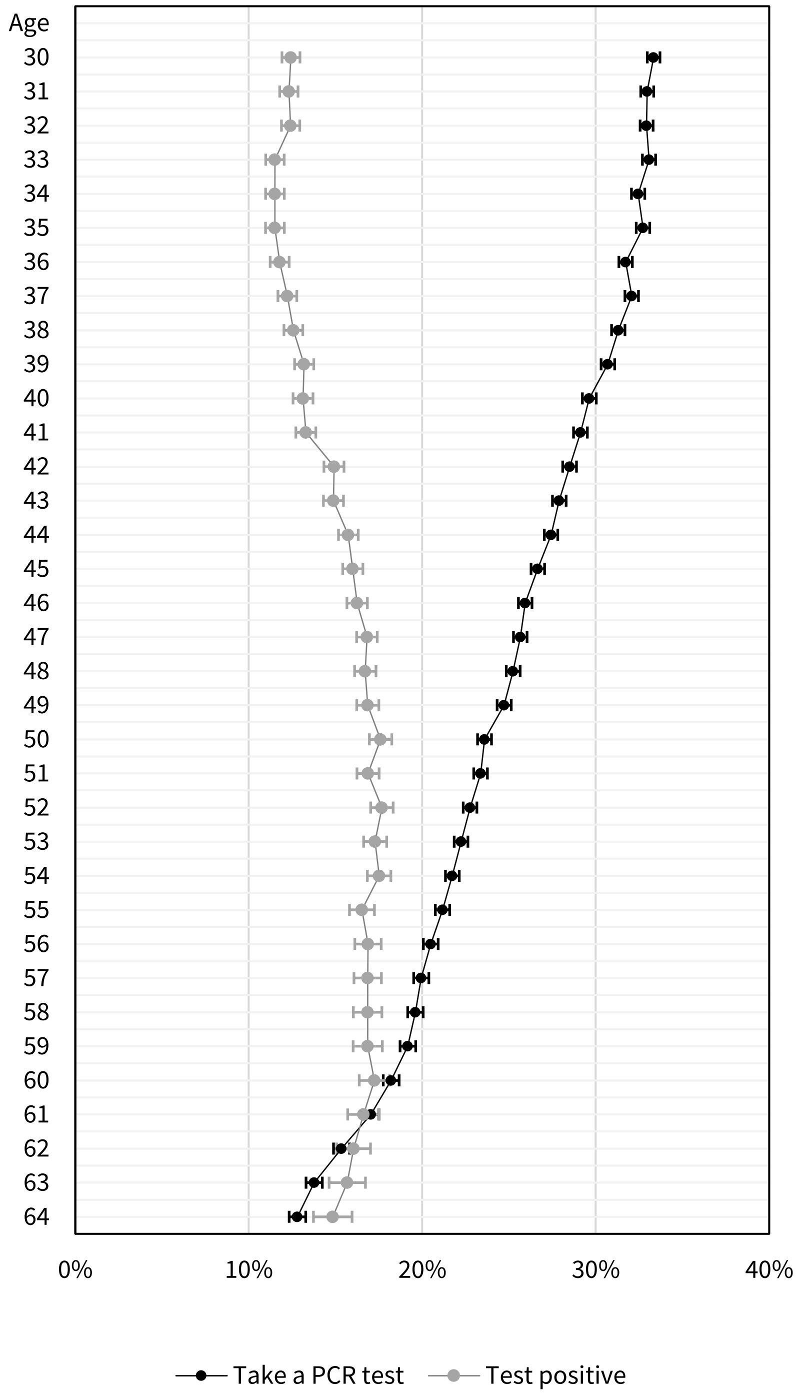

Note: The probability of taking a test is estimated in the full analytical sample (n=1 480 126) and the probability of testing positive is estimated in individuals in the sample that took at least one PCR test (n=384 638). Predictive margins are estimated by fixing the variable of interest at a specific value for all individuals while keeping the other variables at their observed values, predicting the value of the dependent variable for all individuals and then averaging those predictions.

Figure S2. Predictive margins for taking a PCR-test and testing positive by occupations classified by 1-digit (larger markers with 95% confidence intervals) and 3-digit (smaller markers) SSYK-codes in Stockholm and Scania, Sweden, 2020-07-01—2020-12-31.

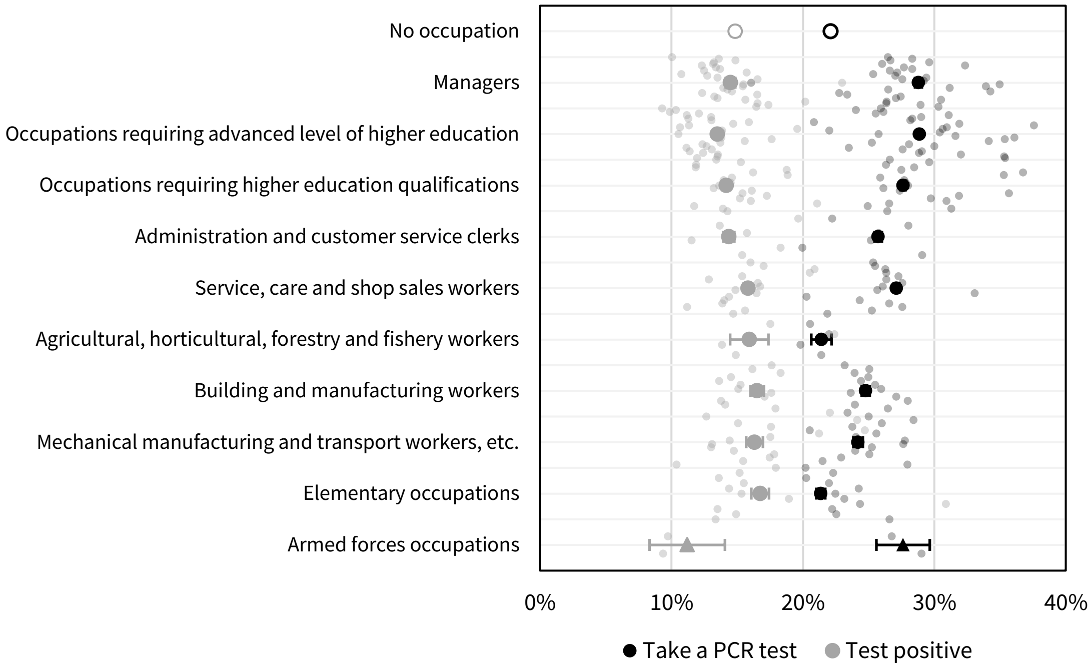

Note: Linear regression adjusted for sex, household size, migrant status, education, income, medical risk factors and household members with medical risk factors with fixed effects for DeSO and birth year. The probability of taking a test is estimated in the full analytical sample (n=1 480 126) and the probability of testing positive is estimated in individuals in the sample that took at least one PCR test (n=384 638). No occupation denotes those inactive in the labour market. Predictive margins are estimated by fixing the variable of interest at a specific value for all individuals while keeping the other variables at their observed values, predicting the value of the dependent variable for all individuals and then averaging those predictions.

Figure S3. Mutually adjusted rate ratios for getting hospitalized, taking a PCR test, testing positive and by type of medical risk factor identified in either the individual or in a household member, Stockholm and Scania, Sweden, 2020-07-01—2020-12-31.

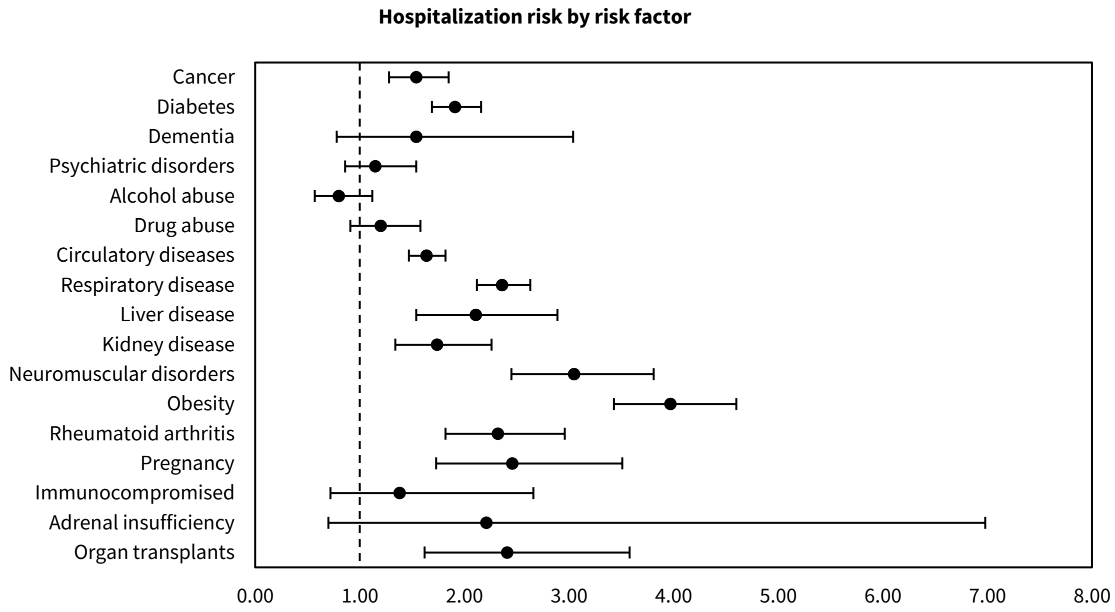

Testing behaviour by risk factor

	Individual		Household member	
	PCR test	Test positive	PCR test	Test positive
Cancer	0.96 (0.94, 0.98)	0.95 (0.90, 1.00)	0.93 (0.91, 0.95)	1.01 (0.96, 1.06)
Diabetes	1.00 (0.98, 1.01)	1.00 (0.97, 1.04)	0.96 (0.95, 0.98)	1.09 (1.05, 1.13)
Dementia	0.44 (0.35, 0.56)	1.24 (0.75, 2.05)	0.73 (0.64, 0.82)	0.89 (0.64, 1.23)
Psychiatric disorders	1.03 (1.00, 1.06)	0.77 (0.71, 0.84)	1.03 (0.99, 1.06)	0.93 (0.84, 1.02)
Alcohol abuse	0.94 (0.90, 0.97)	0.82 (0.74, 0.91)	0.99 (0.96, 1.02)	1.01 (0.93, 1.10)
Drug abuse	0.82 (0.79, 0.97)	0.68 (0.59, 0.77)	0.98 (0.95, 1.02)	1.06 (0.98, 1.16)
Circulatory diseases	1.09 (1.09, 1.10)	0.96 (0.94, 0.98)	0.96 (0.95, 0.97)	1.06 (1.03, 1.08)
Respiratory disease	1.25 (1.24, 1.26)	0.87 (0.84, 0.89)	1.09 (1.08, 1.09)	1.00 (0.98, 1.02)
Liver disease	0.97 (0.91, 1.03)	0.88 (0.75, 1.05)	1.01 (0.95, 1.07)	0.99 (0.85, 1.16)
Kidney disease	0.86 (0.82, 0.90)	0.92 (0.81, 1.06)	0.93 (0.89, 0.97)	1.06 (0.96, 1.18)
Neuromuscular disorders	0.88 (0.85, 0.92)	0.95 (0.86, 1.05)	0.95 (0.92, 0.98)	0.95 (0.88, 1.03)
Obesity	1.12 (1.10, 1.14)	0.96 (0.91, 1.01)	1.06 (1.04, 1.08)	0.95 (0.91, 1.00)
Rheumatoid arthritis	1.08 (1.04, 1.11)	0.90 (0.83, 0.99)	0.98 (0.95, 1.01)	1.00 (0.92, 1.08)
Pregnancy	0.93 (0.92, 0.95)	0.98 (0.92, 1.04)	1.10 (1.09, 1.12)	0.85 (0.80, 0.89)
Immunocompromised	0.98 (0.91, 1.05)	0.84 (0.68, 1.05)	1.04 (1.00, 1.09)	0.90 (0.78, 1.04)
Adrenal insufficiency	0.96 (0.83, 1.12)	1.13 (0.76, 1.70)	0.99 (0.86, 1,15)	0.98 (0.66, 1.47)
Organ transplants	0.88 (0.79, 0.97)	1.00 (0.76, 1.30)	0.90 (0.82, 1.00)	1.05 (0.82, 1.34)

Note: Not having the specific medical risk factor is the reference category for each rate ratio. Poisson regression adjusted for sex, household size, migration background, education and income with fixed effects DeSO, occupation and birth year. The probability of taking a test is estimated in the full analytical sample (n=1 480 126) and the probability of testing positive is estimated in individuals in the sample that took at least one PCR test (n=384 638).

Figure S4. Predictive margins and 95% confidence intervals for taking a PCR-test and testing positive by demographic, socioeconomic and medical factors in Stockholm and Scania, Sweden, 2020-07-01—2020-12-31 by calendar month. The probabilities are estimated using mutually adjusted linear regression with fixed effects for DeSO, birth year and occupation in 148 categories. The estimates were allowed to vary by month were obtained by introducing two-way interaction terms between all included covariates and calendar month. To account for the same individual being observed at several time points, clustered standard errors were used where each individual was defined as a cluster. The probability of taking a test is estimated in the full analytical sample (n=1 480 126) and the probability of testing positive is estimated in individuals in the sample that took at least one PCR test (n=384 638). Predictive margins are estimated by fixing the variable of interest at a specific value for all individuals while keeping the other variables at their observed values, predicting the value of the dependent variable for all individuals and then averaging those predictions. The figure continues on the next page.

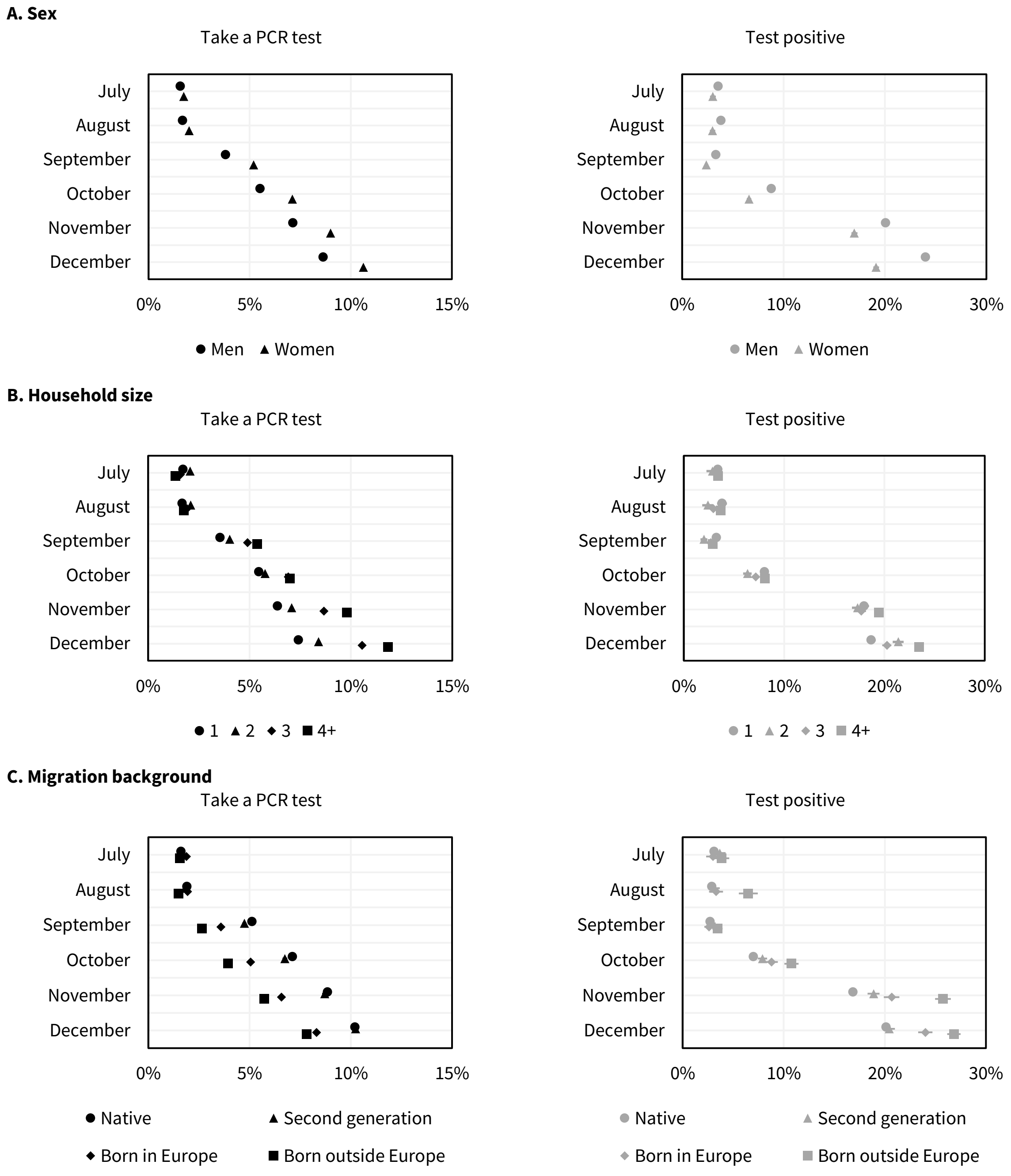

D. Education

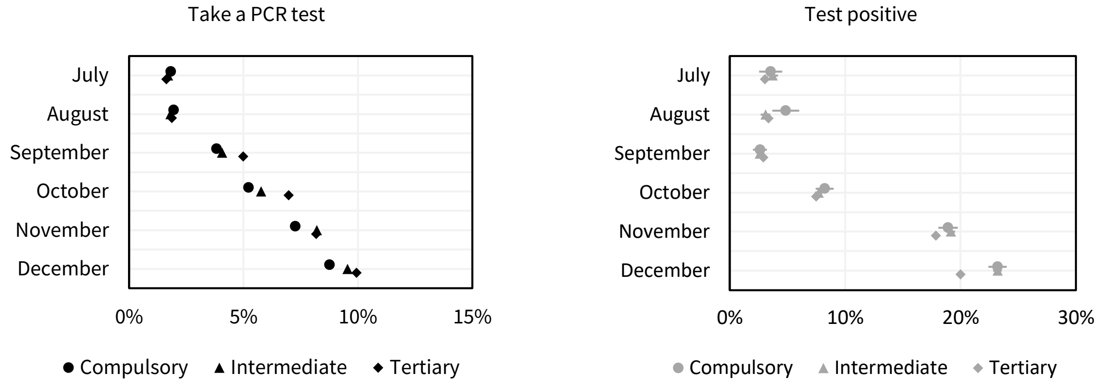

E. Income

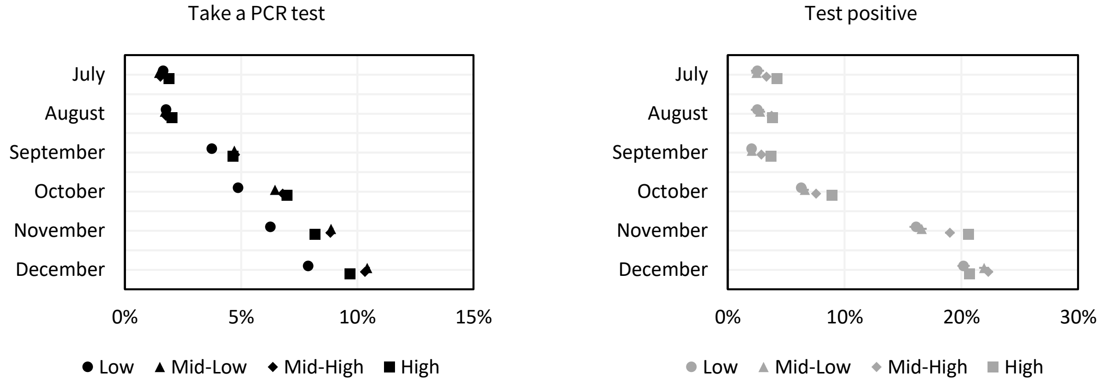

F. Medical risk factor (individual)

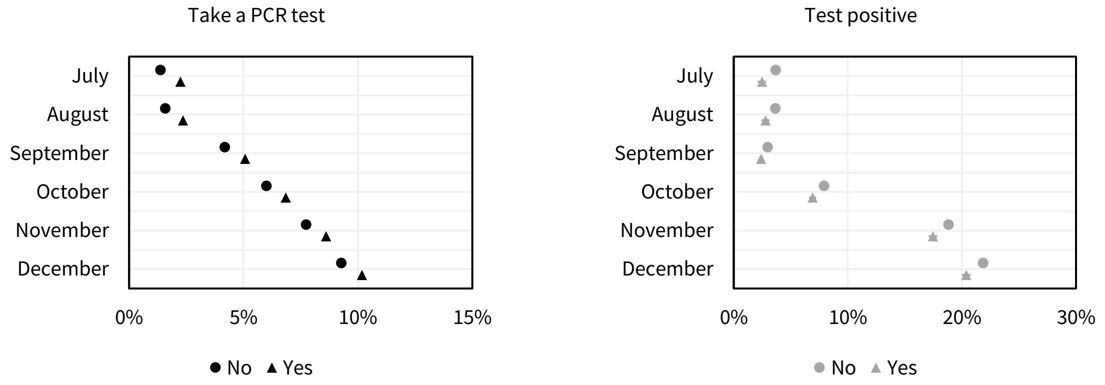

G. Medical risk factor (Household)

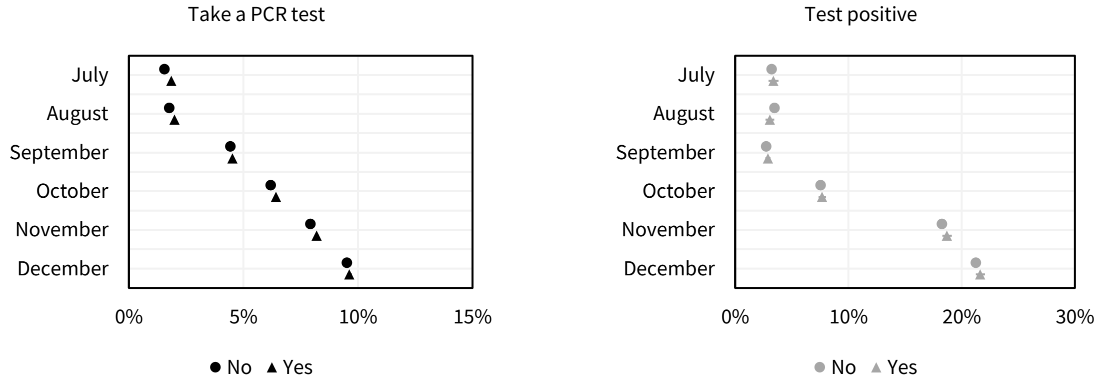

Table S3. Number of tests and positive tests by calendar month among persons aged 30-64, not employed in health or social care, resident in Stockholm and Scania, Sweden during 2020-07-01—2020-12-31.

Month	PCR tests	Positive tests
July	26 178	833
August	28 226	913
September	68 516	1713
October	98 063	7392
November	122 986	22 597
December	150 730	32 023

Figure S5. Number of individuals taking multiple tests and positivity rate by number of tests taken and incidence rate ratios and 95% confidence intervals for PCR testing by number of tests taken in Stockholm and Scania, Sweden, 2020-07-01—2020-12-31.

A. Number of individuals taking multiple tests and positivity rates by number of taken tests

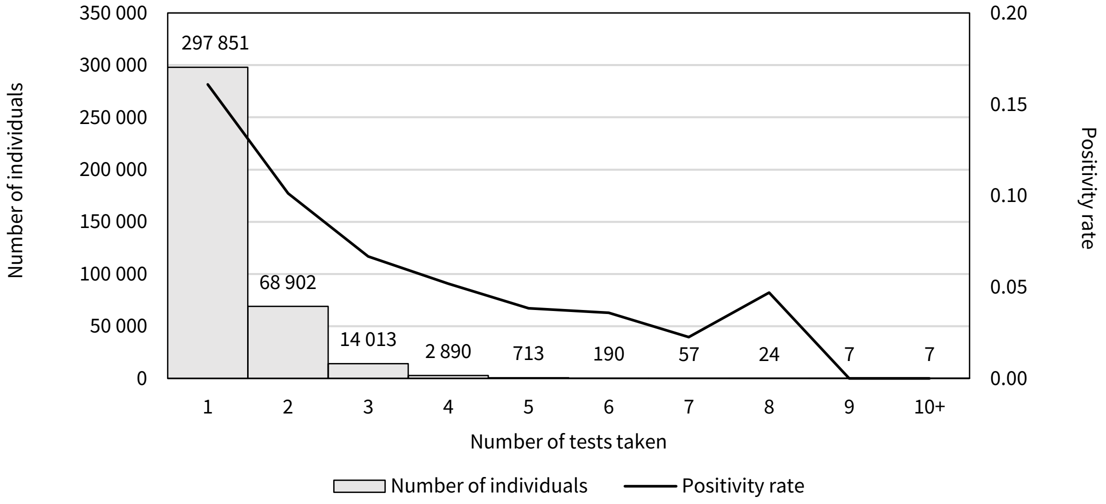

B. Incidence rate ratios for PCR testing by numbr of tests taken

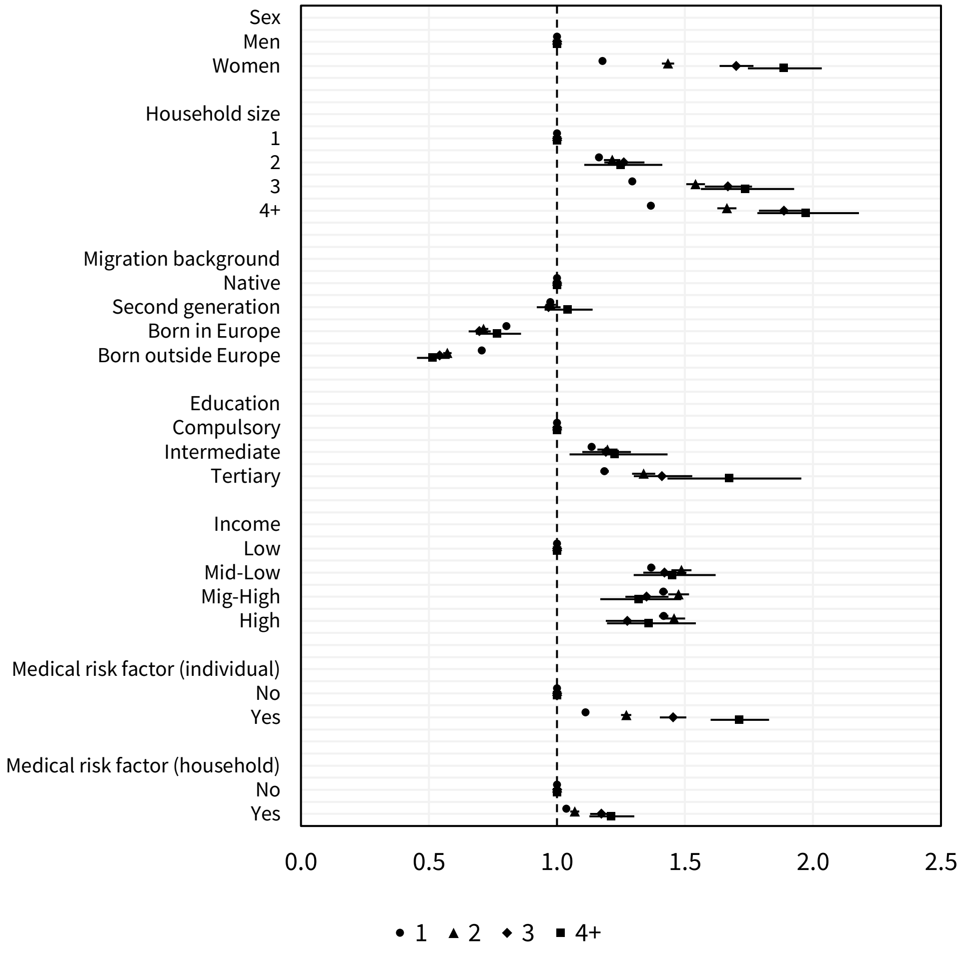

Note: Relative risks were estimated using fixed-effects Poisson models. The models included all presented covariates as well as with fixed effects for age, DeSO and occupation.

Table S4. Alternative model specifications. Regression coefficients and standard errors for taking a PCR test and test positivity, Stockholm and Scania, Sweden during 2020-07-01—2020-12-31. Linear regression with fixed effects. The table continues on the next page.

	Take a PCR test				Test positive			
	Main	Uni	No DeSO	RF Sep.	Main	Uni	No DeSO	RF Sep.
Sex								
Men	0.000	0.000	0.000	0.000	0.000	0.000	0.000	0.000
Women	0.049	0.062	0.049	0.050	-0.032	-0.036	-0.034	-0.035
Household size								
1	0.000	0.000	0.000	0.000	0.000	0.000	0.000	0.000
2	0.026	0.048	0.027	0.031	0.002	-0.001	0.001	0.001
3	0.062	0.084	0.064	0.064	0.003	0.003	0.003	0.004
4+	0.082	0.107	0.082	0.081	0.025	0.026	0.024	0.024
Migration background								
Native	0.000	0.000	0.000	0.000	0.000	0.000	0.000	0.000
Second generation	-0.008	-0.015	-0.009	-0.008	0.010	0.011	0.013	0.010
Born in Europe	-0.056	-0.093	-0.062	-0.056	0.027	0.026	0.038	0.027
Born outside Europe	-0.085	-0.118	-0.097	-0.085	0.065	0.067	0.081	0.064
Education								
Missing	-0.058	-0.121	-0.056	-0.060	-0.015	-0.005	-0.019	-0.016
Compulsory	0.000	0.000	0.000	0.000	0.000	0.000	0.000	0.000
Intermediate	0.020	0.051	0.023	0.019	-0.005	-0.012	-0.008	-0.005
Tertiary	0.035	0.090	0.042	0.034	-0.024	-0.037	-0.027	-0.024
Income quartile								
Low	0.000	0.000	0.000	0.000	0.000	0.000	0.000	0.000
Mid-Low	0.061	0.106	0.062	0.059	0.007	0.003	0.005	0.007
Mid-High	0.067	0.124	0.070	0.065	0.020	0.009	0.015	0.019
High	0.065	0.115	0.069	0.062	0.024	0.009	0.019	0.023
Risk factor (individual)								
No	0.000	0.000	0.000		0.000	0.000	0.000	
Yes	0.033	0.035	0.032		-0.013	-0.017	-0.014	
Risk factor (household)								
No	0.000	0.000	0.000		0.000	0.000	0.000	
Yes	0.008	0.040	0.007		0.003	0.009	0.002	
Risk factor (individual)								
Cancer				-0.012				-0.008
Diabetes				0.005				0.001
Dementia				-0.054				0.032
Psychiatric disorders				0.006				-0.028
Alcohol abuse				-0.010				-0.024
Drug abuse				-0.031				-0.046
Circulatory diseases				0.021				-0.006
Respiratory disease				0.065				-0.019
Liver disease				-0.003				-0.017
Kidney disease				-0.027				-0.011
Neuromuscular disorders				-0.026				-0.006
Obesity				0.029				-0.006
Rheumatoid arthritis				0.017				-0.014
Pregnancy				-0.014				0.004
Immunocompromised				-0.009				-0.018
Adrenal insufficiency				-0.010				0.010
Organ transplants				-0.023				-0.005
Risk factor (household)								
Cancer				-0.023				0.002
Diabetes				-0.011				0.016
Dementia				-0.049				-0.022
Psychiatric disorders				0.005				-0.012
Alcohol abuse				-0.006				0.001

	Take a PCR test				Test positive			
	Main	Uni	No DeSO	RF Sep.	Main	Uni	No DeSO	RF Sep.
Drug abuse				-0.009				0.010
Circulatory diseases				-0.017				0.009
Respiratory disease				0.029				-0.001
Liver disease				-0.001				0.003
Kidney disease				-0.015				0.009
Neuromuscular disorders				-0.015				-0.008
Obesity				0.009				-0.008
Rheumatoid arthritis				-0.004				0.001
Pregnancy				0.032				-0.023
Immunocompromised				0.021				-0.015
Adrenal insufficiency				-0.008				-0.004
Organ transplants				-0.022				0.012
Fixed effects								
Age	Yes	Yes	Yes	Yes	Yes	Yes	Yes	Yes
Occupation	Yes	No	Yes	Yes	Yes	No	Yes	Yes
DeSO	Yes	Yes	No	Yes	Yes	Yes	No	Yes
N	1 480 126	1 480 126	1 480 126	1 480 126	384 638	384 638	384 638	384 638

Note: Main=reference model (represented as predictive margins in Figure 1), Uni=univatriate models, No DeSO=excluding DeSO fixed effects, RF Sep=Risk factors modelled separately. The coefficient for each risk factor denotes the difference between having the risk factor and not having the risk factor in the individual or household member respectively.
